# Exploring the role of artificial intelligence in chemotherapy development, cancer diagnosis, and treatment: present achievements and future outlook

**DOI:** 10.3389/fonc.2025.1475893

**Published:** 2025-02-04

**Authors:** Bassam Abdul Rasool Hassan, Ali Haider Mohammed, Souheil Hallit, Diana Malaeb, Hassan Hosseini

**Affiliations:** ^1^ Department of Pharmacy, Al Rafidain University College, Baghdad, Iraq; ^2^ School of Pharmacy, Monash University Malaysia, Subang Jaya, Malaysia; ^3^ School of Medicine and Medical Sciences, Holy Spirit University of Kaslik, Jounieh, Lebanon; ^4^ Department of Psychology, College of Humanities, Effat University, Jeddah, Saudi Arabia; ^5^ Applied Science Research Center, Applied Science Private University, Amman, Jordan; ^6^ College of Pharmacy, Gulf Medical University, Ajman, United Arab Emirates; ^7^ Institut Coeur et Cerveau de l’Est Parisien (ICCE), UPEC-University Paris-Est, Creteil, France; ^8^ RAMSAY SANTÉ, Hôpital Paul D’Egine (HPPE), Champigny sur Marne, France

**Keywords:** artificial intelligence (AI), machine learning (ML), deep learning (DL), chemotherapy development, cancer diagnosis, cancer treatment

## Abstract

**Background:**

Artificial intelligence (AI) has emerged as a transformative tool in oncology, offering promising applications in chemotherapy development, cancer diagnosis, and predicting chemotherapy response. Despite its potential, debates persist regarding the predictive accuracy of AI technologies, particularly machine learning (ML) and deep learning (DL).

**Objective:**

This review aims to explore the role of AI in forecasting outcomes related to chemotherapy development, cancer diagnosis, and treatment response, synthesizing current advancements and identifying critical gaps in the field.

**Methods:**

A comprehensive literature search was conducted across PubMed, Embase, Web of Science, and Cochrane databases up to 2023. Keywords included “Artificial Intelligence (AI),” “Machine Learning (ML),” and “Deep Learning (DL)” combined with “chemotherapy development,” “cancer diagnosis,” and “cancer treatment.” Articles published within the last four years and written in English were included. The Prediction Model Risk of Bias Assessment tool was utilized to assess the risk of bias in the selected studies.

**Conclusion:**

This review underscores the substantial impact of AI, including ML and DL, on cancer diagnosis, chemotherapy innovation, and treatment response for both solid and hematological tumors. Evidence from recent studies highlights AI’s potential to reduce cancer-related mortality by optimizing diagnostic accuracy, personalizing treatment plans, and improving therapeutic outcomes. Future research should focus on addressing challenges in clinical implementation, ethical considerations, and scalability to enhance AI’s integration into oncology care.

## Introduction

Artificial intelligence (AI) has been extensively applied across multiple medical fields, marking a transformative impact on diverse therapeutic areas, including ophthalmology, radiology, and dermatology. The integration of AI technologies into fundamental biology, pharmacology, and clinical medicine has triggered significant enhancements in performance, achieving benchmarks on that match or exceed human experts’ performance in specific domains (1, 2).

The potential of AI to revolutionize cancer research, diagnosis, and treatment is particularly notable given its advanced analytical capacities. The proliferation of large-scale cancer research datasets presents an unprecedented opportunity to amalgamate intricate research insights with vast data arrays, necessitating robust computational power to manage and interpret these complex information streams (1, 2).

Cancer, one of the most severe illnesses, remains the second leading cause of mortality worldwide, with its prevalence continuing to rise despite global efforts to combat it (1, 2). Current tools and procedures for early-stage cancer detection and diagnosis often lack accuracy, leaving many cases undiagnosed. Recent technological advancements in combinatorial chemistry, genomics, and proteomics have made many databases of biological and chemical data easily accessible. These advancements significantly enhance our understanding of cancer biology at the molecular level, which is critical for improving cancer identification and management in clinical settings (1, 2). The vast quantity of unprocessed molecular data currently available poses a challenge for clinical oncologists in identifying therapeutically significant information. Due to the limited capacity of the human mind to process large amounts of data rapidly, AI has experienced significant growth in the last decade. This growth demonstrates the potential of AI as a platform for generating highly solid decisions (1, 2).

While AI is rapidly being integrated into oncologic research, the advancement of AI solutions is still in its nascent phase (1, 2). Only a limited number of AI-based applications, specifically those employed by healthcare facilities and drug manufacturers, have been granted certification for broader commercial utilization. The question of whether AI can replace medical doctors as professionals is still a matter of ongoing debate. The public discourse surrounding AI applications in cancer clinical research has focused significantly on their development (1, 2). Recent studies underscore AI’s transformative potential in cancer care, from improving diagnostic accuracy to personalizing treatment plans. Dehingia et al. (2022) explored AI’s role in cancer control, emphasizing its impact on medical imaging and predictive modeling. Similarly, Xu et al. (2019) demonstrated AI’s ability to predict lung cancer treatment responses using deep learning models, highlighting its clinical relevance. Moreover, recent advancements in Edge AI (Dehingia et al., 2021) show promise in enhancing accessibility and scalability in cancer management systems. These contributions emphasize the need for continued exploration of AI applications in oncology to address gaps in treatment efficacy and patient outcomes (3–5).

**Figure 1:**
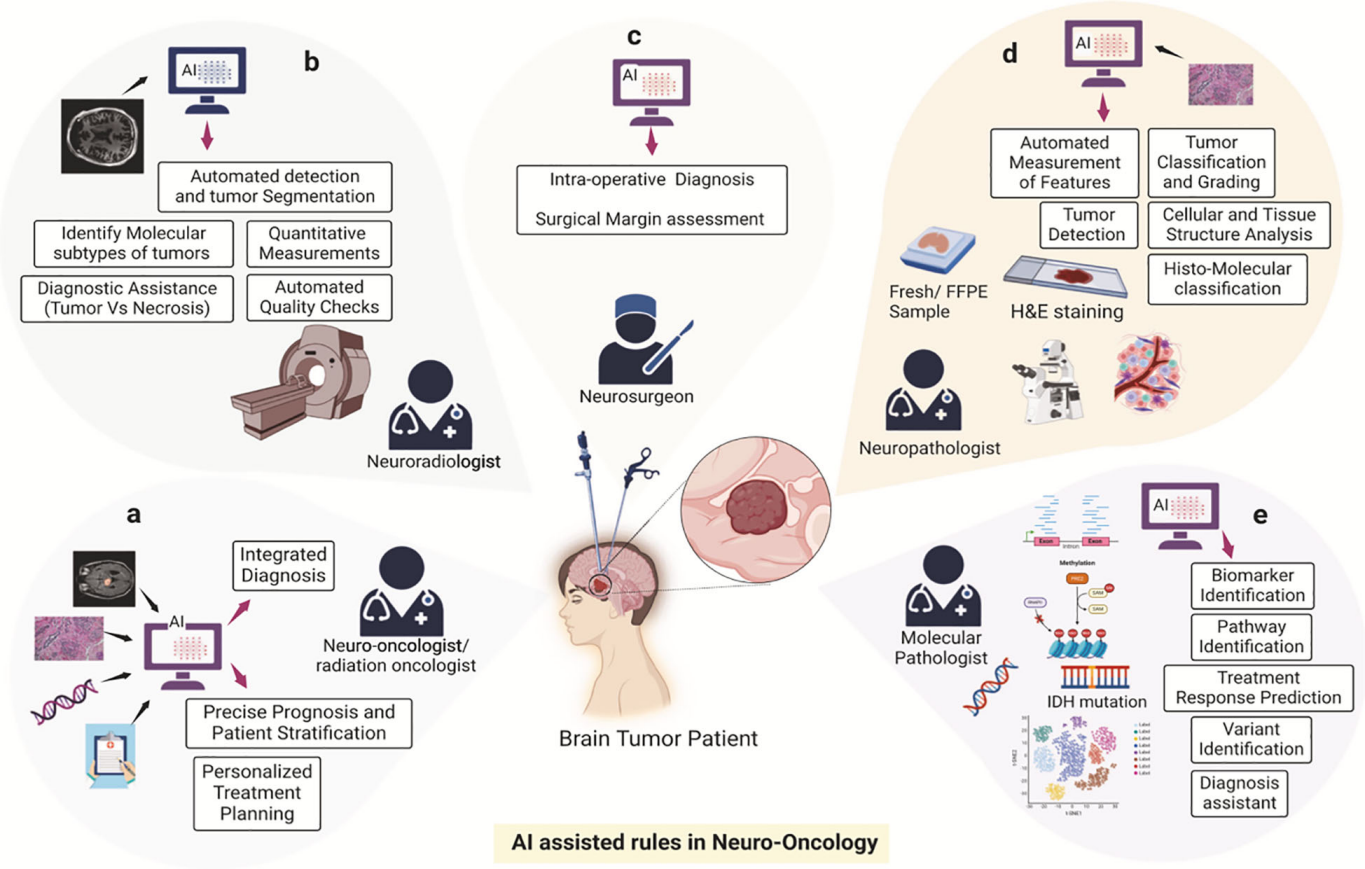
AI-assisted framework in oncology. This schematic illustrates the transformative role of Artificial Intelligence (AI) in cancer care, encompassing chemotherapy development, diagnosis, and treatment planning. The components include: **(A)** Imaging data integration to enhance diagnostic accuracy. **(B)** Histopathological data interpretation for personalized treatment strategies. **(C)** Genomic and molecular data analysis to identify therapeutic targets. **(D)** Clinical data aggregation for optimizing treatment workflows. **(E)** Predictive modeling for therapeutic response and toxicity mitigation. Reproduced from Khalighi et al., “Artificial intelligence in neuro-oncology: advances and challenges in brain tumor diagnosis, prognosis, and precision treatment,” npj Precision Oncology, 2024, under the Creative Commons Attribution 4.0 International License (https://creativecommons.org/licenses/by/4.0/).

For example, **Figure 1** illustrates the multifaceted role of Artificial Intelligence (AI) in transforming cancer care, encompassing chemotherapy development, cancer diagnosis, and treatment planning. The schematic emphasizes AI’s capacity to integrate diverse datasets—spanning imaging, histopathology, genomics, and clinical data—to enhance diagnostic accuracy, personalize treatment plans, and predict therapeutic responses. By automating data interpretation, identifying molecular patterns, and optimizing workflows, AI facilitates innovative approaches to streamline oncology practices. This integrated framework exemplifies the potential of AI to improve patient outcomes through precision medicine, as demonstrated by its application across various domains in neuro-oncology (4).

This review presents collected empirical evidence on the role of artificial intelligence (AI) in the domain of cancer diagnosis, research, and treatment.

## Methods

We systematically searched the PubMed, Embase, Web of Science, and Cochrane databases up to 2023 using the term “Artificial Intelligence (AI)” and its subset “machine learning (ML)” combined with the following terms: “chemotherapy development”, “cancer treatment”, and “cancer response to treatment” to capture recent trends in AI applications. Our search included review and research articles published within the last 4 years and originally published in English (3, 4).

### Artificial intelligence and cancer drug development

Artificial intelligence (AI) can forecast the effectiveness of anticancer drugs and aid in developing new treatments. Tumors and medications often show varied responses, and high-throughput screening reveals the link between genetic heterogeneity in cancer cells and treatment efficacy (5). High throughput screening (HTS) is the use of automated equipment to rapidly test thousands to millions of samples for biological activity at the model organism, cellular, pathway, or molecular level. In its most common form, HTS is an experimental process in which 10^3^–10^6^ small molecule compounds of known structure are screened in parallel. Sanjeevi and Li (2022) highlight that AI technologies, including machine and deep learning, significantly enhance cancer diagnosis and treatment development. However, creating models for complex tumors remains challenging due to the lack of effective treatments and reliable computational tools. The later study explores advanced AI techniques in anticancer drug discovery, focusing on molecular docking and interaction analysis to facilitate drug discovery. The results emphasized AI’s role in utilizing imaging, molecular, and cellular data to design and discover cancer drugs accelerating anticancer treatment discovery with a reduction in the costs (6).

Similarly, Alex and Peter developed classification models using recent screening data and machine learning to predict compound activity against cancer cells. Based on the mutational state of 145 oncogenes and chemical descriptors, these models have an Area Under the Curve of 0.94, 87% sensitivity, and 87% specificity. Additionally, regression models predicting log half maximal inhibitory concentration values show robust performance, with Pearson correlations of 0.86 in cross-validation. Even with incomplete screening data, their models maintain classification accuracy, offering efficient and implementable screening technologies for personalized oncology, drug repurposing, and discovery (7).

In line with these advancements, Na Ly Tran et al. (2023) discuss AI’s potential in next-generation drug development to find cancer targets and new uses for drugs. As a result, N-(1-propyl-1H-1,3-benzodiazol-2-yl)-3-(pyrrolidine-1-sulfonyl) benzamide (Z29077885) was selected as a new cancer-fighting drug. Tests in both *in-vitro* and *in-vivo* confirmed its effectiveness against different types of cancer by blocking STK33, encouraging apoptosis, and stopping the cell cycle. This AI-driven approach underscores the promise of AI-repurposed drugs for cancer treatment (8).

Caroline et al. (2024) further argue that AI could revolutionize preclinical drug development and clinical trial design. AI enhances tumor profiling, molecular pathway definition, and novel treatment development. Their review highlights AI’s role in molecular screening, target identification, and the creation of analogues with specified characteristics. Moreover, AI combined with CRISPR technology identifies new targets and predicts resistance mechanisms showcasing its transformative potential in drug discovery (9).

### Artificial intelligence and cancer treatment and response

In the domain of cancer treatment, particularly chemotherapy, artificial intelligence (AI) focuses on analyzing the interactions between medications and patients, achieving effective administration, predicate patient tolerance and response, and optimizing treatment plans (5). Many cancer-related deaths result from medication resistance leading to treatment failure even in the early stages. Current therapy strategies based on cancer subtypes and genetic mutations vary in predictive power, necessitating AI-based prediction algorithms to match patients with effective drugs. New developments in AI, especially machine learning have made prediction models better in preclinical settings. However, the lack of clinically relevant pharmacogenomic data makes it harder to develop models that can be used in clinical settings, even though computers are faster (10). Atousa et al. (2021) used NCBI Gene Expression Omnibus database data to predict which cancer cell lines would be resistant to cisplatin. They used the Fisher Score method to choose features and machine learning algorithms to find out the samples that were resistant and sensitive. Their analysis revealed several genes associated with chemoresistance, paving the way for further investigations (11). Similarly, Yue Wang and colleagues demonstrated that cancer cell lines can replicate the lncRNA transcriptomic, genomic, and epigenetic changes observed in tumors. Using LENP models, they accurately predicted drug sensitivities across 21 cancer types, showcasing AI’s potential in treatment response prediction (12). Using a similar method, Xiaoyu et al. (2024) created deep learning models to guess how cells will react to replication stress-inducing agents. These models found molecular complexes that affect drug sensitivity and resistance, which led to new insights into precision medicine (13). Addressing chemotherapy toxicity, Agata and colleagues developed CURATE.AI, an AI-powered platform that dynamically determines optimal chemotherapy doses using minimal patient-specific data. Their clinical trial demonstrated a significant dose reduction with improved patient response rates, highlighting AI’s role in enhancing treatment efficacy and supporting further trials (14).

#### AI and breast cancer

Ornella and Caterina (2024) discussed the use of artificial intelligence (AI) and machine learning to analyze vast data for personalized medicine, emphasizing the challenges of gathering extensive biological data and developing predictive models. They concentrated on triple-negative breast cancer (TNBC), a disease currently lacking targeted treatments but under exploration for novel therapies such as immunotherapy. Given immunotherapy’s success with melanoma, the researchers evaluated its potential for TNBC, noting that AI and predictive techniques could be beneficial. The principles of immune system stimulation, checkpoint inhibitors, and individualized treatments used in melanoma could enhance outcomes for TNBC patients, offering new hope for this hard-to-treat cancer (15). Similarly, Lixuan et al. (2023) created a machine learning model to analyze unstructured clinical electronic health record (EHR) data for predicting recurrence probability in breast cancer patients post-surgery. A total of 1,841 patients were analyzed and key traits were extracted linked to recurrence risk from clinical notes and histology reports demonstrating the model’s utility for personalized treatment planning (16). In a related study, Karen et al. (2022) utilized clinical and pathological data from 130 patients to develop AI models predicting the response to neoadjuvant chemotherapy. Their artificial neural network was very good at predicting pathologic complete response, locoregional recurrence, and vital status. This shows that AI has the potential to predict different outcomes in breast cancer patients (17). Moreover, Zhi and colleagues introduced the IMage-based Pathological REgistration and Segmentation Statistics (IMPRESS) pipeline, an automated feature extraction workflow for whole slide images (WSIs), comparing H&E and multiplex IHC images to predict outcomes of neoadjuvant chemotherapy in HER2-positive and TNBC patients. Their AI-based approach outperformed manually created features, particularly for Human Epidermal Growth Factor-2 positive (HER2+) subtype, with external validation by other cohorts (18). To improve the low pathological complete response (pCR) rates in early breast cancer patients who were positive for hormone receptors but negative for HER2, Luca et al. (2023) created machine learning models using clinical and pathological data. Their random forests algorithm performed best, accurately predicting pCR and associating it with longer disease-free survival, thus helping tailor treatment (19). Similarly, Savitri et al. (2023) developed an AI model using a deep convolutional neural network to predict NAC response in TNBC. Their model autonomously extracted morphometric characteristics from digitized tissue images, showing significant predictive potential and aiding personalized treatment decisions (20). Recent advancements in breast cancer treatment have not established a definitive factor predicting NAC susceptibility for locally advanced cases. This was the reason why a study came up with a new AI pipeline method that used three separate models to guess how preoperative chemotherapy would affect needle biopsies stained with hematoxylin and eosin. This combined model achieved 95.15% accuracy in predicting NAC response, indicating its potential to facilitate personalized medicine in NAC therapy for breast cancer (21).

#### AI and colorectal cancer

As the third most common cancer globally, colorectal cancer presents significant clinical challenges (22). Beyond traditional surgical and chemotherapy methods, newly discovered molecular processes offer broader treatment options. However, selecting personalized treatments remains difficult in the age of precision medicine. Recently, research into artificial intelligence (AI) for colorectal cancer treatment has surged, focusing on AI’s potential in managing the disease. After a thorough literature search in MEDLINE, EMBASE, and Web of Science, 49 publications were included, demonstrating AI’s ability to predict treatment outcomes and provide crucial guidance for both conventional and innovative therapies, enabling personalized treatment plans for colorectal cancer patients. Machine learning and deep learning techniques have shown favorable results in evaluating prognoses and selecting treatment strategies for colorectal cancer patients (22). Valentina et al. (2022) employed AI to create prognostic and predictive models for therapy response, focusing on activity/efficacy and toxicity, to aid clinical decision-making. This systematic review assessed AI’s efficacy in predicting chemotherapy responses, alone or with targeted therapy in metastatic colorectal cancer (mCRC) patients. A total of 26 original papers were retrieved through Medline publications from April 2022 which met the inclusion criteria. They found that different algorithms were very good at predicting how therapy would work, with delta-radiomics and 74 gene signatures were good at telling the difference between responders and non-responders with up to 99% accuracy for responders and 100% accuracy for non-responders. This research suggests that AI can develop individualized treatment protocols for mCRC, and identify comprehensive markers that predict both efficacy and toxicity which is highly valuable for future clinical trials (23). Similarly, Zugang et al. (2023) emphasized the importance of neoadjuvant chemoradiotherapy (NCRT) in treating colorectal cancer (CRC), particularly rectal cancer. Intermediate-risk patients often receive adjuvant chemotherapy, but most do not require further treatment, making accurate clinical decision-making vital. AI integration into therapy decision-making and efficacy evaluation benefits NCRT patients, while clinical decision support systems (CDSSs) powered by AI reduce medical errors. South Korean experts developed a CRC chemotherapy recommender based on real data achieving satisfactory accuracy with an AUC > 0.95. Despite its specific and solitary data source, it marks a significant advancement. To provide new risk classifications post-colectomy, researchers also created deep learning (DL) CDSSs and the DoMorev1-CRC marker, allowing low-risk patients to avoid unnecessary NCRT and improving survival rates. Clinicians use prognostic assessments of CRC patients to choose the best treatments. Current research is focusing on using DL-assisted MRI to predict metastases in locally advanced rectal cancer (LARC) patients who are undergoing NCRT. Fernando et al. created a classifier that accurately predicted drug resistance (AUC = 0.93) by combining stable biomarkers like lncRNAs with a lot of computing power (24). Additionally, Watson for Oncology (WFO) from IBM provides personalized, evidence-based treatment strategies for colorectal cancer. Batuer et al. (2021) compared WFO’s recommendations with those from a multidisciplinary team (MDT) at a Shanghai cancer center, studying 250 stage II–IV colorectal cancer patients treated between March 2017 and January 2018. Concordance was defined if MDT’s decisions matched WFO’s “recommended” or “for consideration” categories, with the study finding 91% overall concordance and rates varying by stage and treatment method. After updating the WFO database to address discrepancies, Concordance improved, with the authors concluding that WFO’s recommendations were highly similar to the MDT’s, suggesting that AI decision-support systems like WFO can enhance precision medicine in oncology (25). Furthermore, Ferrari et al. conducted a retrospective study to develop and validate an AI model using texture analysis of high-resolution T2-weighted MR images to predict treatment response in LARC patients. The AI model looked at 55 patients who had a 3T MRI before, during, and after neoadjuvant chemoradiotherapy (CRT). The goal was to predict both pathologic complete response (CR) and non-responders (NR). Textural features were extracted using open-source software, with significant features selected and AI models trained and validated on separate cohorts. The CR prediction AI model had an AUC of 0.86, and the NR model had an AUC of 0.83. This showed that the AI models were better than standard care and that AI models based on MR image texture can accurately predict complete response and non-response in LARC patients early in the treatment process (26).

#### AI and lung cancer

Colton et al. (2023) compiled a comprehensive literature analysis on artificial intelligence (AI) in the context of lung cancer, highlighting its implications for interdisciplinary care teams. Their findings demonstrated that various data points collected throughout a patient’s diagnosis and treatment are crucial for optimizing outcomes through precision medicine. By utilizing existing data sets such as molecular information and radiomics, along with patient and tumor characteristics, AI can aid in developing models that detect cancer early and provide personalized treatment plans (27). Building on this, Pranali and colleagues emphasized the transformative potential of AI in lung cancer management, from screening to treatment. They noted that AI-driven radiomic models effectively distinguish between benign and malignant lung nodules, while advanced deep learning models like Sybil predicts future lung cancer risk using low-dose CT scans. AI also aids in predicting the efficacy of targeted therapies and immunotherapies, and in active surveillance by identifying high-risk recurrence factors (28). Another review discussed AI mechanism in lung cancer diagnosis, treatment, and prognosis. This could lower death rates by figuring out patients at risk, confirming the disease, and predicting treatment effectiveness by combining EHR records, imaging studies, histopathology reports, and molecular biomarkers (29). Yue et al. (2022) underscored the benefits of combining AI, radiomics, and genomic data with clinical data to improve patient care, while also noting challenges such as limited racial diversity in AI model testing and the opaque nature of neural networks (30). Samantha discussed the ongoing challenges in AI implementation for lung cancer treatment, emphasizing the need for standardized principles and guidelines, and the role of machine learning (ML) in enhancing healthcare efficiency and solving complex problems in cancer research (31). Additionally, Lawek et al. (2021) demonstrated that machine learning is effective for early lung cancer detection and individualized treatment plans. They developed predictive models trained on 39 variables using data from the IASLC staging project, emphasizing the need for further validation with larger prospective studies, even though ML enhances clinical decision-making (32).

#### AI and ovarian cancer

Deanna and her colleagues (2022) looked into whether AI can find morphologic patterns in high-grade serious ovarian cancer (HGSOC) during laparoscopy and connect its effect on improving patient’s health. They analyzed 435 still-frame photos from 113 patients with confirmed HGSOC (2013-2019). Using Deep Learning (DenseNet 201), they trained an AI model with 70% of images for training, 10% for validation, and 20% for testing. The model distinguished between patients with favorable (ER: progression-free survival ≥12 months) and unfavorable (PR: survival ≤6 months) responses. For ER patients, the AI achieved 93% accuracy and 100% sensitivity, but had 63% specificity which misclassifys some PR patients. This highlights AI’s potential in predicting HGSOC outcomes from surgical images and refining clinical treatment plans (33). Doga et al. (2019) introduced SigMA, a novel computational technique for accurately detecting the HR deficient mutational signature from targeted gene panels, expanding the pool of patients eligible for therapies aimed at HR insufficiency by facilitating panel-based identification of mutational signatures, crucial for those with BRCA1/2 mutations and similar patterns (34). Qingyi and colleagues (2023) assessed the predictive utility of machine learning for responses to platinum-based chemotherapy in ovarian cancer patients. They found that various models, including support vector machines, showed high accuracy in predicting therapy responses and guiding future scoring system development (35). Yanlia et al. (2024) reviewed AI’s role in extracting high-throughput data from medical and pathological images, significantly enhancing the diagnosis and prognostic assessment of ovarian cancer (OC), with AI-based radiomics being particularly valuable in gynecological settings (36). Munetoshi and Kazunori (2020) used AI to predict the pathology diagnosis of ovarian tumors by analyzing patient records and preoperative exam data, finding AI effective in predicting ovarian cancer pathology using preoperative exams (37). A study that looked at AI-based ovarian cancer diagnostic and prognostic studies using histopathology data showed that AI models can do tasks like stain quantification, histological subtyping, and treatment response prediction tool but many models are not yet ready for use in the real world (38). Sian et al. (2024) conducted a review on the use of AI in ovarian malignancy detection via ultrasound. They found that AI demonstrated good diagnostic performance, but further prospective research is necessary to confirm its usefulness in healthcare settings (39). Meixuan and colleagues (2024) created an understandable machine learning model for figuring out the diagnosis and prognosis of EOC using biomarkers. This showed that ML was better than traditional methods at predicting outcomes and placing medicine as a more precise treatment option (40). He-Li et al. (2022) conducted a comprehensive literature review on AI’s diagnostic performance in ovarian cancer using medical imaging, demonstrating that AI systems performed well in OC diagnosis, although future studies require stricter reporting criteria (41). Finally, Raoof (2024) demonstrated the predictive efficiency of machine learning (ML) for screening high-risk OC groups, suggesting that ML approaches offer significant potential for OC screening and preventive strategies (42).

#### AI and prostate cancer

Artificial intelligence (AI) has demonstrated potential in the detection of prostate cancer in biopsies, yet the outcomes have been largely limited to individual research endeavors without global substantiation. Competitions like the Prostate cANcer graDe Assessment (PANDA) challenge, organized by Wouter et al. (2022), have expedited advancements in medical imaging, though their effectiveness is often hindered by a lack of reproducibility and independent validation. The PANDA challenge, the most extensive histopathology competition thus far, attracted 1,290 developers and utilized 10,616 digitized prostate specimens to stimulate the creation of AI algorithms for Gleason grading. The challenge confirmed that many submitted algorithms achieved performance comparable to that of pathologists, showing agreements with expert uropathologists on both U.S. and European validation sets. This underscores the potential for AI-based Gleason grading to generalize across diverse populations and laboratories, warranting further prospective clinical trials (43). Expanding on this, Indrani et al. (2022) reviewed AI’s role in assisting radiologists, pathologists, and urologists in prostate cancer management, noting that AI models for imaging and histopathology images perform admirably but require more research for robust and generalizable applications. AI’s ability to reduce variability in Gleason grading and streamline clinical workflows is promising, yet integration into clinical practice needs more exploration (44). With advancements in medical science, we are entering a “big data” era where multidimensional datasets are crucial for medical modeling. AI approaches like machine learning (ML) and deep learning (DL) algorithms can process these datasets, reducing inconsistencies and improving clinical data representation. This is particularly important in prostate cancer, where AI can enhance diagnosis, risk assessment, and patient surveillance by minimizing subjectivity and facilitating decision-making (45). Additionally, AI-powered chatbots like prostate cancer communication assistant (PROSCA), developed by Magdalena et al. (2023), demonstrate the potential of AI in enhancing patient education, improving knowledge, and supporting clinical practice (46). Ronan et al. (2019) assessed the effects of AI in managing prostate cancer (PCa) through genetic testing, imaging diagnostics, and pathology which can improve treatment options and outcome prediction (47). James and Julian (2024) observed that AI advancements in prostate cancer treatment reflect broader progress in healthcare and information technology, with AI improving diagnostic imaging, pathology, and therapy outcome forecasting and enhancing efficiency in radiation oncology (48). The integration of AI and robotics in prostate cancer care is revolutionary offering early diagnosis and personalized treatment plans while addressing challenges like data privacy and algorithm biases. This synergy between AI and robotics promises improved diagnostics, personalized treatments, and research advancements, heralding a new era in PCa management (49).

#### AI and brain cancer

Review research by Sirvan et al. (2024) explores the latest developments in neuro-oncology’s use of artificial intelligence (AI), particularly focusing on gliomas a type of brain tumor that poses a major threat to global health. AI has revolutionized brain tumor management by integrating imaging, histological, and genomic data streamlining processes such as diagnosis, classification, prognosis, and treatment planning. AI models have proven to be more accurate and specific than human evaluators in diagnosing, predicting, and treating malignant brain tumors. They can detect molecular features in images, potentially shortening the time to molecular diagnosis and reducing the need for invasive procedures. Current applications and challenges include deep learning and classical machine learning approaches, integrating data from various sources, developing medical language models, addressing gender and racial inequalities, and accurately delineating and characterizing tumors. Adaptive personalized therapy approaches are also prioritized for maximizing therapeutic outcomes. Social, ethical, and legal ramifications are discussed, promoting transparency and equity in AI use in neuro-oncology, highlighting its transformative impact on patient care (4). Tumor classification, an essential for targeted treatment, has seen improvements through AI and machine learning, which have demonstrated promising results in automatic segmentation and classification using imaging modalities like MRI and CT (50). Maurizio et al. (2023) noted that AI is accelerating the transition to patient-specific brain tumor treatment, impacting diagnostics and treatment processes. Radiomics enables non-invasive, repeatable characterization of lesions, aiding treatment planning, while AI-powered instruments in surgical planning improves precision in brain surgery. AI models also predict complications, recurrences, and therapeutic responses which enhance follow-up strategies and risk stratification (51). Anil et al. (2023) highlighted AI’s role in enhancing cancer imaging interpretation, tumor volume delineation, and genotyping, with AI-assisted brain surgery being a safe and effective option. They emphasized overcoming obstacles to fully realize AI’s potential in healthcare (52). Simon and colleagues reviewed AI’s impact on brain tumor surgery management from pre-operative screening to post-operative care which emphasizes the importance of collaboration in creating AI training datasets. This mandatory open access to machine learning algorithms, and adherence to reporting criteria in clinical trials exerts vital role. Despite AI’s potential, there are concerns about the reduction of skills among physicians and job displacement, underscoring the need for careful AI integration in neurosurgery (53).

#### AI and pancreatic cancer

Detection of pancreatic cancer is challenging as it is associated with a terrible prognosis due to its aggressive nature and the pancreas difficult location which complicate access. The lack of early signs and specific markers often delay diagnosis. While advancements in imaging have facilitated diagnosis where refining standards for best practices remains essential. The heterogeneity of tumors further complicates histological examination and biopsies. Artificial intelligence (AI) is transforming healthcare by enhancing diagnosis, treatment, and patient care. AI systems can accurately analyze medical images aiding early disease detection and supporting personalized medicine by analyzing patient data to create tailored treatment plans while streamlining administrative tasks like medical coding and documentation. In this context, Satvik et al. (2024) highlight AI’s potential to revolutionize pancreatic cancer care by improving diagnoses, providing personalized treatments, and streamlining operations ultimately benefiting patients. The authors emphasize AI’s significant potential to enhance patient outcomes through early detection, precise diagnosis, treatment selection, and prognosis prediction. However, to ensure the effective and ethical use of AI in pancreatic cancer treatment, we must address issues such as data accessibility, model interpretability, and ethical concerns (54). Similarly, Bahrudeen and Uma (2022) noted the advantages of AI in diagnosing pancreatic cancer with imaging technologies but highlighted the lack of substantial datasets and ethical concerns as barriers to widespread clinical implementation (55). Bowen et al. underscored the importance of early detection and treatment of pancreatic cancer noting AI’s role in identifying high-risk groups and predicting recurrence, metastasis, therapy response, and survival (56). Guohua et al. (2024) discussed the need for ethical guidelines and standards to protect patient confidentiality and data integrity in AI applications, emphasizing the importance of collaboration between clinicians and researchers (57). AI’s transformative potential in pancreatic cancer is further evidenced by its ability to analyze extensive patient data and medical images, facilitating earlier and more precise diagnoses and treatment decisions (54). Hai-Min et al. (2020) highlighted AI’s role in overcoming treatment challenges such as drug resistance and surgical complexity, as well as improving radiotherapy and robotic surgery precision (58). Christoph et al. reviewed AI’s role in pancreatic surgery noting its potential in pre-, intra-, and postoperative diagnostics, decision-making, and training, and emphasized the need for robust collaboration between surgeons and data scientists to advance AI applications (59). Lastly, Kwang-Sig et al. (2021) demonstrated AI’s potential as a decision-support system in clinical practice by analyzing recurrence in pancreatic cancer post-surgery, though further research is needed to establish its tangible benefits (60).

#### AI and thyroid cancer

There is a growing prevalence of thyroid nodules being detected each year increasing the likelihood of unnecessary treatments or incorrect diagnoses. The study by Maksymilian et al. (2023) provides the latest insights into the use of artificial intelligence (AI) for diagnosing and classifying thyroid nodules highlighting the advanced applications of AI in ultrasonography for diagnosing and characterizing pathology. Their analysis of 930 publications, narrowed down to 33 key studies, underscores the significant potential of AI in the future categorization and diagnosis of thyroid nodules, extending beyond cancer differentiation to innovative uses (61). Despite advancements in diagnostic, surgical, and chemotherapy techniques, the diagnosis and treatment of thyroid cancer remain complex, as noted by Lakshmi et al. (2023). They pointed out challenges such as inaccuracies in predicting outcomes, uncertainties in diagnosing cell abnormalities, and inconsistencies in ultrasound image interpretation. AI-assisted algorithms have shown substantial improvements in sensitivity and specificity in thyroid ultrasonography and cytopathology compared to conventional methods, although limited clinical experience and the need for prospective validation studies remain key barriers. Prospective, multi-center trials are essential for integrating AI into precision medicine for thyroid cancer (62). Nan et al. (2022) highlighted the necessity of precise preoperative diagnosis for effective thyroid cancer surgery. They introduced a machine learning system that outperformed human judgment in predicting thyroid nodule malignancy, demonstrating AI’s superiority in preoperative diagnostics (63). Ling-Rui and colleagues further explored AI’s role in enhancing personalized medicine for thyroid cancer. Their review focused on AI’s ability to extract and analyze morphological, textural, and molecular data, providing insights for tailored treatments. They emphasized that addressing challenges in ultrasonic and pathological testing through AI can facilitate the implementation of individualized treatment protocols (64).

#### AI and leukemia

Artificial intelligence (AI) and machine learning (ML) have permeated every industry in the modern era, significantly impacting the field of cancer research, particularly in the detection and treatment of leukemia. Leukemia, or blood cancer, is among the most prevalent hematological diseases and is notoriously difficult to detect early, making it challenging to treat due to a scarcity of effective medications. Consequently, modernizing diagnostic instruments and methods is crucial. AI and ML technologies have garnered considerable interest for their role in predictive medicine and contemporary oncological practices. Hematologic malignancies, including leukemia, are often undiagnosed until they progress to an incurable stage, emphasizing the importance of early detection and accurate diagnosis. Through automated detection models, AI improves diagnostic accuracy and reduces clinicians’ workload. For instance, a study utilizing the InceptionV3 model for acute lymphocytic leukemia (ALL) classification achieved 98.38% accuracy, outperforming other transfer learning approaches and demonstrating AI’s potential in medical diagnostics (65). In 2021, Min et al. showed that AI could be useful for sorting blood cells, using convolutional neural networks (CNNs) to do a great job of diagnosing ALL and other conditions (66). Basel et al. (2023) emphasized the need for further investigation into AI-driven techniques like CNNs for ALL diagnosis from bone marrow imaging, noting their higher sensitivity and specificity compared to peripheral blood smears (67). Mustafa et al. (2021) considered machine learning models for finding and classifying leukemia using images of peripheral blood smears as an accurate, quick, and low-cost diagnostic services (68). Nathan and colleagues discussed the expanding role of ML in analyzing complex genetic, epigenetic, immunologic, and regulatory pathways in hematological disorders, which is crucial for understanding and treating these diseases (69). Jan-Niklas et al. (2020) reviewed advancements in ML techniques for managing acute myeloid leukemia (AML), emphasizing the importance of collaboration among scientists, researchers, and clinicians to ensure secure and effective ML integration in medical practice (70). Marian et al. (2022) introduced explainable AI (XAI) through a method called multi-dimensional module optimization (MOM), providing interpretable and robust predictions for AML treatment, thus enhancing the comprehension of AI medical judgments (71). These advancements underscore the transformative potential of AI and ML in improving the diagnosis, treatment, and management of leukemia, although further research and validation are necessary to fully integrate these technologies into clinical practice (72).

#### AI and lymphoma

The battle against lymphatic cancer relies heavily on early detection and accurate diagnosis, with artificial intelligence (AI) showing potential promise in medical imaging. Anying et al. (2024) conducted a comprehensive review and meta-analysis of AI’s diagnostic performance in detecting lymphoma through medical imaging. using Medline, Embase, Institute of Electrical and Electronics Engineers, and Cochrane up until December 2023. The study revealed that AI models achieved a pooled sensitivity of 87%, specificity of 94%, and an area under the curve (AUC) of 97%, suggesting strong diagnostic capabilities. Despite these promising results, the authors highlighted the need for additional studies with stricter standards to avoid overestimation (73). Concurrently, Joaquim et al. (2024) emphasized the multidisciplinary consensus among hematologists, geneticists, and clinicians for the categorization of lymphoid neoplasms, incorporating clinical observations, histological traits, immunophenotype, and molecular pathology. This agreement uses AI’s power to handle large datasets to improve the detection and diagnosis of mature B-cell tumors by using different neural networks and machine learning techniques. AI’s role in incorporating cell-of-origin markers, such as CD3, CD5, CD19, and others, underscores its growing significance in medical diagnostics (74). Over the past decade, the digitalization of medical records and multi-omics data analysis has facilitated access to high-dimensional datasets, allowing AI and machine learning (ML) to emerge as vital tools in lymphoma pathology. Junyun et al. (2024) noted that AI and digital mining have transitioned lymphoma pathology analysis from qualitative to quantitative, significantly improving diagnostic accuracy and objectivity. These advancements in ML techniques not only enhance lymphoma diagnostics but also contribute to better prognosis and more personalized treatment plans, illustrating the potential and limitations of ML in clinical practice (75, 76).

## Conclusion

Artificial intelligence (AI), including its subsets of machine learning (ML) and deep learning (DL), has demonstrated significant potential in transforming chemotherapy development, cancer diagnosis, and treatment. This scoping review highlights AI’s role in improving diagnostic accuracy, optimizing treatment plans, and predicting patient responses, ultimately contributing to reduced cancer-related mortality. However, despite these advancements, challenges remain in integrating AI solutions into clinical practice.

Future research should focus on addressing critical gaps such as the incorporation of pharmacogenomic and multi-omics data into AI algorithms, which could enhance the precision of personalized medicine. Longitudinal studies are needed to validate the clinical utility of AI-driven predictions and interventions across diverse populations. Furthermore, developing scalable, cost-effective AI systems tailored for resource-limited settings can ensure equitable access to advanced cancer care globally.

## Data Availability

The original contributions presented in the study are included in the article/supplementary material. Further inquiries can be directed to the corresponding authors.

## References

[1] Sufyan M Shokat Z Ali Ashfaq U . Artificial intelligence in cancer diagnosis and therapy: Current status and future perspective. Comput Biol Med. (2023) 165:107356. doi: 10.1016/j.compbiomed.2023.107356 37688994

[2] Weerarathna IN Kamble AR Luharia A . Artificial intelligence applications for biomedical cancer research: A review. Cureus. (2023) 15:e48307. doi: 10.7759/cureus.48307 38058345 PMC10697339

[3] Dehingia K Jeelani MB Das A . Artificial intelligence and machine learning: A smart science approach for cancer control. In: Advances in Deep Learning for Medical Image Analysis, vol. 2022. CRC Press, Taylor & Francis Group, Boca Raton, FL (2022).

[4] Khalighi S Reddy K Midya A Pandav KB Madabhushi A Abedalthagafi M . Artificial intelligence in neuro-oncology: advances and challenges in brain tumor diagnosis, prognosis, and precision treatment. NPJ Precis Oncol. (2024) 8:80. doi: 10.1038/s41698-024-00575-0 38553633 PMC10980741

[5] Xu Y Hosny A Zeleznik R Parmar C Coroller T Franco I . Deep learning predicts lung cancer treatment response from serial medical imaging. Clin Cancer Res. (2019) 25 (11):3266–75. doi: 10.1158/1078-0432.CCR-18-2495 PMC6548658 31010833

[6] Pandiyan S Wang Li . A comprehensive review on recent approaches for cancer drug discovery associated with artificial intelligence. Comput Biol Med. (2022) 150:106–40. doi: 10.1016/j.compbiomed.2022.106140 36179510

[7] Lind AP Anderson PC . Predicting drug activity against cancer cells by random forest models based on minimal genomic information and chemical properties. PloS One. (2019) 14:e0219774. doi: 10.1371/journal.pone.0219774 31295321 PMC6622537

[8] Tran NaLy Kim H Shin Cheol−Hee Ko E Oh SJa . Artifcial intelligence-driven new drug discovery targeting serine/threonine kinase 33 for cancer treatment. Cancer Cell Int. (2023) 23:1–11. doi: 10.1186/s12935-023-03176-2 38087254 PMC10717841

[9] Bailleux C Gal J Chamorey E Mograbi B Milano Gérard . Artificial intelligence and anticancer drug development keep a cool head. Pharmaceutics. (2024) 16:1–6. doi: 10.3390/pharmaceutics16020211 PMC10893490 38399265

[10] Rafique R Islam R Kazi JU . Machine learning in the prediction of cancer therapy. Comput Struct Biotechnol J. (2021) 19:4003–17. doi: 10.1016/j.csbj.2021.07.003 PMC8321893 34377366

[11] Ataei A Majidi NS Zahiri J Rostami M Arab SS Rizvanov AA . Prediction of chemoresistance trait of cancer cell lines using machine learning algorithms and systems biology analysis. J Big Data. (2021) 8:1–21. doi: 10.1186/s40537-021-00477-z 33425651

[12] Wang Y Wang Z Xu J Li J Li S Zhang M . Systematic identification of non-coding pharmacogenomic landscape in cancer. Nat Commun. (2018) 9:3192. doi: 10.1038/s41467-018-05495-9 30093685 PMC6085336

[13] Zhao X Singhal A Park S Kong J Bachelder R Ideker T . Cancer mutations converge on a collection of protein assemblies to predict resistance to replication stress. Cancer Discovery. (2024) 14:508–23. doi: 10.1158/2159-8290.CD-23-0641 PMC10905674 38236062

[14] Blasiak A Truong A Jeit W Tan L Kumar KS Tan SB . PRECISE CURATE.AI: A prospective feasibility trial to dynamically modulate personalized chemotherapy dose with artificial intelligence. J Clin Oncol. (2022) 40:1574. doi: 10.1200/JCO.2022.40.16_suppl.1574 35157496

[15] Garrone O La Porta CAM . Artificial intelligence for precision oncology of triple-negative breast cancer: learning from melanoma. Cancers. (2024) 16:1–13. doi: 10.3390/cancers16040692 PMC10887240 38398083

[16] Zeng L Liu L Chen D Lu H Xue Y Bi H . The innovative model based on artificial intelligence algorithms to predict recurrence risk of patients with postoperative breast cancer. Front Oncol. (2023) 13:1117420. doi: 10.3389/fonc.2023.1117420 36959794 PMC10029918

[17] Goulart KOB Kneubil MC Brollo J Orlandin BC Corso LL Roesch-Ely M . Use of artificial intelligence to predict response to neoadjuvant chemotherapy in breast cancer. Mastology. (2023) 33:e20220041. doi: 10.29289/Z25945394

[18] Huang Z Shao W Han Z Alkashash AM Sancha Cdela Parwani AV . Artificial intelligence reveals features associated with breast cancer neoadjuvant chemotherapy responses from multi-stain histopathologic images. NPJ Precis Oncol. (2023) 7:1–15. doi: 10.1038/s41698-023-00352-5 36707660 PMC9883475

[19] Mastrantoni L Garufi G Maliziola N Monte EDi Arcuri G Frescura V . Artificial intelligence (AI) –based machine learning models (ML) for predicting pathological complete response (pCR) in patients with hormone receptor (HoR) –positive/HER2- negative early breast cancer (EBC) undergoing neoadjuvant chemotherapy (NCT): A retrospective cohort study. J Clin Oncol. (2023) 41:597. doi: 10.1200/JCO.2023.41.16_suppl.597

[20] Krishnamurthy S Jain P Tripathy D Basset R Randhawa R Muhammad H . Predicting response of triple-negative breast cancer to neoadjuvant chemotherapy using a deep convolutional neural network–based artificial intelligence tool. Am Soc Clin Oncol. (2023) 7:1–11. doi: 10.1200/CCI.22.00181 PMC10530970 36961981

[21] Shen B Saito A Ueda Ai Fujita K Nagamatsu Y Hashimoto M . Development of multiple AI pipelines that predict neoadjuvant chemotherapy response of breast cancer using H&E-stained tissues. J Pathol: Clin Res. (2023) 9:182–94. doi: 10.1002/cjp2.v9.3 PMC10073928 36896856

[22] Yang J Huang J Han D Ma X . Artificial intelligence applications in the treatment of colorectal cancer: A narrative review. Clin Med Insights: Oncol. (2024) 18:1–17. doi: 10.1177/11795549231220320 PMC10771756 38187459

[23] Russo V Lallo E Munnia A Spedicato M Messerini L D’Aurizio R . Artificial intelligence predictive models of response to cytotoxic chemotherapy alone or combined to targeted therapy for metastatic colorectal cancer patients: a systematic review and meta-analysis. Cancers. (2022) 14:4012. doi: 10.3390/cancers14164012 36011003 PMC9406544

[24] Yin Z Yao C Zhang L Qi S . Application of artificial intelligence in diagnosis and treatment of colorectal cancer: A novel Prospect. Front Med. (2023) 10:1128084. doi: 10.3389/fmed.2023.1128084 PMC10030915 36968824

[25] Aikemu B Xue P Hong H Jia H Wang C Li S . Artificial intelligence in decision-making for colorectal cancer treatment strategy: an observational study of implementing watson for oncology in a 250-case cohort. Front Oncol. (2021) . 10:594182. doi: 10.3389/fonc.2020.594182 33628729 PMC7899045

[26] Ferrari R Mancini-Terracciano C Voena C Rengo M Zerunian M Ciardiello A . MR-based artificial intelligence model to assess response to therapy in locally advanced rectal cancer. Eur J Radiol. (2019) 18:1–9. doi: 10.1016/j.ejrad.2019.06.013 31439226

[27] Ladbury C Amini A Govindarajan A Mambetsariev I Raz DJ Massarelli E . Integration of artificial intelligence in lung cancer: Rise of the machine. Cell Rep Med. (2023) 4:1–11. doi: 10.1016/j.xcrm.2023.100933 PMC9975283 36738739

[28] Pachika P Valasapalli S Ngo P Kloecker G . The use of artificial intelligence in lung cancer management. AI Precis Oncol. (2024) 1:33–42. doi: 10.1089/aipo.2023.0002

[29] Pei Q Luo Y Chen Y Li J Xie D Ye T . Artificial intelligence in clinical applications for lung cancer: diagnosis, treatment and prognosis. Clin Chem Lab Med. (2022) 60:1974–83. doi: 10.1515/cclm-2022-0291 35771735

[30] Wang Y Cai H Pu Y Li J Yang F Yang C . The value of AI in the diagnosis, treatment, and prognosis of Malignant lung cancer. Front Radiol. (2022) 2:1–12. doi: 10.3389/fradi.2022.810731 PMC10365105 37492685

[31] Williams S . Use of artificial intelligence for lung cancer treatment. Int J Advancements Technol. (2021) 12:128.

[32] Berzenji L Debaenst S Yogeswaran S Lauwers P Hendriks JM Van Schil PE . Predicting the future: using AI to predict treatment outcomes in lung cancer. J Thorac Oncol. (2021) 16:192. doi: 10.1016/j.jtho.2021.01.079

[33] Glassman D Handley K Fleming N Westin S Jazaeri A Rauh-Hain J . How to train your robot: Artificial intelligence predicts treatment response in ovarian cancer. Gynecologic Oncol. (2022) 166:S44. doi: 10.1016/S0090-8258(22)01286-0

[34] Gulhan DC Lee JJ-K Melloni GEM Cortés-Ciriano I Park PJ . Detecting the mutational signature of homologous recombination deficiency in clinical samples. Nat Genet. (2019) 51:912–9. doi: 10.1038/s41588-019-0390-2 30988514

[35] Wang Q Chang Z Liu X Wang Y Feng C Ping Y . Predictive value of machine learning for platinum chemotherapy responses in ovarian cancer: systematic review and meta-analysis. J Med Internet Res. (2024) 26:e48527. doi: 10.2196/48527 38252469 PMC10845031

[36] Wang Y Lin W Zhuang X Wang X He Y Li L . Advances in artificial intelligence for the diagnosis and treatment of ovarian cancer (Review). Oncol Rep. (2024) 51:1–17. doi: 10.3892/or.2024.8705 38240090 PMC10828921

[37] Akazawa M Hashimoto K . Artificial intelligence in ovarian cancer diagnosis. Anticancer Res. (2020) 40:4795–800. doi: 10.21873/anticanres.14482 32727807

[38] Breen J Allen K Zucker K Adusumilli P Scarsbrook A Hall G . Artificial intelligence in ovarian cancer histopathology: a systematic review. NPJ Precis Oncol. (2023) 7:1–14. doi: 10.1038/s41698-023-00432-6 37653025 PMC10471607

[39] Mitchell S Nikolopoulos M El-Zarka A Al-Karawi D Al-Zaidi S Ghai A . Artificial intelligence in ultrasound diagnoses of ovarian cancer: a systematic review and meta-analysis. A Systematic Review and Meta-Analysis. Cancers. (2024) 16:1–12. doi: 10.3390/cancers16020422 PMC10813993 38275863

[40] Wu M Zhao Y Dong X Jin Y Cheng S Zhang N . Artificial intelligence-based preoperative prediction system for diagnosis and prognosis in epithelial ovarian cancer: A multicenter study. . Front Oncol. (2022) 12:975703. doi: 10.3389/fonc.2022.975703 36212430 PMC9532858

[41] Xu H-L Gong T-T Liu F-H Chen H-Y Xiao Q Hou Y . Artificial intelligence performance in image-based ovarian cancer identification: A systematic review and meta-analysis. E Clin Med. (2022) 53:101662. doi: 10.1016/j.eclinm.2022.101662 PMC9486055 36147628

[42] Nopour R . Screening ovarian cancer by using risk factors: machine learning assists. Biomed Eng Online. (2024) 23:1–19. doi: 10.1186/s12938-024-01219-x 38347611 PMC10863117

[43] Bulten W Kartasalo K Chen P-HC Ström P Pinckaers H Nagpal K . Artificial intelligence for diagnosis and Gleason grading of prostate cancer: the PANDA challenge. Nat Med. (2022) 28:154–63. Available online at: www.nature.com/naturemedicine (Accessed September 17, 2024). 10.1038/s41591-021-01620-2 PMC8799467 35027755

[44] Bhattacharya I Khandwala YS Vesal S Shao W Yang Q Soerensen SJC . A review of artificial intelligence in prostate cancer detection on imaging. Ther Adv Urol. (2022) 14:1–31. doi: 10.1177/17562872221128791 PMC9554123 36249889

[45] Rabaan AA Bakhrebah MA AlSaihati H Alhumaid S Alsubki RA Turkistani SA . Artificial intelligence for clinical diagnosis and treatment of prostate cancer. Cancers. (2022) 14:5595. doi: 10.3390/cancers14225595 36428686 PMC9688370

[46] Görtz M Baumgärtner K Schmid T Muschko M Woessner P Gerlach A . An artificial intelligence-based chatbot for prostate cancer education: Design and patient evaluation study. Digital Health. (2023) 9:1–11. doi: 10.1177/20552076231173304 PMC10159259 37152238

[47] Thenault R Kaulanjan K Darde T Rioux-Leclercq N Bensalah K Mermier M . The application of artificial intelligence in prostate cancer management—What improvements can be expected? A Systematic Review Appl Sci. (2020) 10:1–25. doi: 10.3390/app10186428

[48] Yu JB Hong JC . AI use in prostate cancer: potential improvements in treatments and patient care. Oncology. (2024) 38:208–9. 10.46883/2024.25921021 38776517

[49] Arigbede O Amusa T Buxbaum SG . Exploring the use of artificial intelligence and robotics in prostate cancer management. Cureus. (2023) 15:e46021. doi: 10.7759/cureus.46021 37900395 PMC10602629

[50] Kaifi R . A review of recent advances in brain tumor diagnosis based on AI-based classification. Diagnostics. (2023) 13:3007. doi: 10.3390/diagnostics13183007 37761373 PMC10527911

[51] Cè M Irmici G Foschini C Danesini GM Falsitta LV Serio ML . Artificial intelligence in brain tumor imaging: A step toward personalized medicine. Curr Oncol. (2023) 30:2673–701. doi: 10.3390/curroncol30030203 PMC10047107 36975416

[52] Philip AK Samuel BA Bhatia S Khalifa SAM El-Seedi HR . Artificial intelligence and precision medicine: A new frontier for the treatment of brain tumors. Life. (2023) 13:1–16. doi: 10.3390/life13010024 PMC9866715 36675973

[53] Williams S Horsfall HL Funnell JP Hanrahan JG Khan DZ Muirhead W . Artificial intelligence in brain tumor surgery—An emerging paradigm. Cancers. (2021) 13:1–25. doi: 10.3390/cancers13195010 PMC8508169 34638495

[54] Tripathi S Tabari A Mansur A Dabbara H Bridge CP Daye D . From machine learning to patient outcomes: A comprehensive review of AI in pancreatic cancer. Diagnostics. (2024) 14:174. doi: 10.3390/diagnostics14020174 38248051 PMC10814554

[55] Hameed BS Krishnan UM . Artificial intelligence-driven diagnosis of pancreatic cancer. Cancers. (2022) 14:5382. doi: 10.3390/cancers14215382 36358800 PMC9657087

[56] Huang B Huang H Zhang S Zhang D Shi Q Liu J . Artificial intelligence in pancreatic cancer. Theranostics. (2022) 12:6931–54. doi: 10.7150/thno.77949 PMC9576619 36276650

[57] Zhao G Chen Xi Zhu M Liu Y Wang Y . Exploring the application and future outlook of Artificial intelligence in pancreatic cancer. Front Oncol. (2024) 14:1345810. doi: 10.3389/fonc.2024.1345810 38450187 PMC10915754

[58] Lin H-M Xue X-F Wang X-G Dang S-C Gu M . Application of artificial intelligence for the diagnosis, treatment, and prognosis of pancreatic cancer. Artif Intell Gastroenterol. (2020) 1:19–29. doi: 10.35712/aig.v1.i1.19

[59] Kuemmerli C Rössler F Berchtold C Frey MC Studier-Fischer A Cizmic A . Artificial intelligence in pancreatic surgery: current applications. J Pancreatol. (2023) 6:74–81. doi: 10.1097/JP9.0000000000000129

[60] Lee K-S Yoon Y-S Jang J-Y Kang CM Yu Y-D Hwang HoK . Usefulness of artificial intelligence for predicting recurrence following surgery for pancreatic cancer: Retrospective cohort study. Int J Surg. (2021) 93:1–6. doi: 10.1016/j.ijsu.2021.106050 34388677

[61] Ludwig M Ludwig Bartłomiej Kaliszewski K . The use of artificial intelligence in the diagnosis and classification of thyroid nodules: anUpdate. Cancers. (2023) 15:1–24. doi: 10.3390/cancers15030708 PMC9913834 36765671

[62] Nagendra L Pappachan JM Fernandez CJ . Artificial intelligence in the diagnosis of thyroid cancer: Recent advances and future directions. Artif Intell Cancer. (2023) 4:1–10. doi: 10.35713/aic.v4.i1.1

[63] Xi NM Wang L Yan C . Improving the diagnosis of thyroid cancer by machine learning and clinical data. Scientifc Rep. (2022) 12:11143. doi: 10.1038/s41598-022-15342-z PMC9249901 35778428

[64] Li L-R Du Bo Liu H-Q Chen C . Artificial intelligence for personalized medicine in thyroid cancer: current status and future perspectives. Front Oncol. (2021) 10:604051. doi: 10.3389/fonc.2020.604051 33634025 PMC7899964

[65] Alaoui YEl Elomri A Qaraqe M Padmanabhan R Taha RY Omri HEl . A review of artificial intelligence applications in hematology management: current practices and future prospects. J Med Internet Res. (2022) 24:e36490. doi: 10.2196/36490 35819826 PMC9328784

[66] Zhou M Wu K Yu L Xu M Yang J Shen Q . Development and evaluation of a leukemia diagnosis system using deep learning in real clinical scenarios. Front Pediatr. (2021) 9:693676. doi: 10.3389/fped.2021.693676 34249819 PMC8264256

[67] Elsayed B Elshoeibi A Elhadary M Badr A Metwalli O Cherif H . Deep learning models for the diagnosis of acute lymphoblastic leukemia from bone marrow images: A comprehensive literature review. Blood. (2023) 142:7184–5. doi: 10.1182/blood-2023-187037

[68] Ghaderzadeh M Asadi F Hosseini A Bashash D Abolghasemi H Roshanpour A . Machine learning in detection and classification of leukemia using smear blood images: A systematic review. Sci Programming. (2021) 2021:1–14. doi: 10.1155/2021/9933481

[69] Radakovich N Cortese M Nazha A . Acute myeloid leukemia and artificial intelligence, algorithms and new scores. Best Pract Res Clin Hematol. (2020) 33:101192. doi: 10.1016/j.beha.2020.101192 PMC7548395 33038981

[70] Eckardt J-N Bornhauser M Wendt K Middeke JM . Application of machine learning in the management of acute myeloid leukemia: current practice and future prospects. Blood Adv. (2020) 4:6077–6085. doi: 10.1182/bloodadvances.2020002997 33290546 PMC7724910

[71] Gimeno M -Ene´riz ESJose´ Villar S Agirre X Prosper F Rubio A . Explainable artificial intelligence for precision medicine in acute myeloid leukemia. Front Immunol. (2022) 13:977358. doi: 10.3389/fimmu.2022.977358 36248800 PMC9556772

[72] Hussain I Andriyas EA Umar MH Saxena AK . Artificial intelligence and machine learning in early diagnosis of hematological Malignancies. Int J Multidiscip Res. (2024) 7:1–7. doi: 10.36948/ijfmr.2024.v06i01.11627

[73] Bai A Si M Xue P Qu Y Jiang Yu . Artifcial intelligence performance in detecting lymphoma from medical imaging: a systematic review and meta-analysis. BMC Med Inf Decision Making. (2024) 24:1–19. doi: 10.1186/s12911-023-02397-9 PMC10775443 38191361

[74] Carreras J Hamoudi R Nakamura N . Artificial intelligence and classification of mature lymphoid neoplasms. Explor Targeted Anti-tumor Ther. (2024) 5:332–48. doi: 10.37349/etat.2024.00221 PMC11090685 38745770

[75] Yuan J Zhang Ya Wang X . Application of machine learning in the management of lymphoma: Current practice and future prospects. DIGITAL Health. (2024) 10:1–12. doi: 10.1177/20552076241247963 PMC11020711 38628632

[76] Kumar V Gaddam M Moustafa A Iqbal R Gala D Shah M . The utility of artificial intelligence in the diagnosis and management of pancreatic cancer. Cureus. (2023) 15:e49560. doi: 10.7759/cureus.49560 38156176 PMC10754023

